# MicroRNA‐410 serves as a candidate biomarker in hypoxic‐ischemic encephalopathy newborns and provides neuroprotection in oxygen‐glucose deprivation‐injured PC12 and SH‐SY5Y cells

**DOI:** 10.1002/brb3.2293

**Published:** 2021-07-31

**Authors:** Qinghong Meng, Peipei Yang, Yuanyuan Lu

**Affiliations:** ^1^ Department of Neonatology Weifang People's Hospital Weifang Shandong China; ^2^ Department of Pediatrics Weifang People's Hospital Weifang Shandong China

**Keywords:** cell survival, diagnosis, hypoxic‐ischemic encephalopathy, microRNA‐410, oxygen‐glucose deprivation, phosphatase and tensin homolog

## Abstract

**Background:**

MicroRNA‐410 (miR‐410) has been found to be deregulated in neonatal hypoxic‐ischemic encephalopathy (HIE). However, the clinical significance and biological function of miR‐410 remain largely elusive. This study aims to investigate the expression and diagnostic performance of miR‐410 in HIE newborns, and explores the neuroprotective effect of miR‐410 in an oxygen‐glucose deprivation (OGD)‐induced cell injury model.

**Methods:**

Expression of miR‐410 was examined using quantitative real‐time PCR, and its diagnostic performance was evaluated using a receiver operating characteristic analysis. We used OGD‐injured PC12 and SH‐SY5Y cells to construct an *in vitro* HIE model. The effect of miR‐410 on OGD‐induced cell injury was analyzed by assessing cell viability and apoptosis. Enzyme‐linked immunosorbent assay was used to evaluate inflammation in cell model. A target gene was assessed according to the luciferase reporter assay.

**Results:**

Serum miR‐410 expression was significantly decreased in HIE newborns and OGD‐injured cell model. The reduced miR‐410 expression served as a biomarker for the diagnosis and progression of HIE. The OGD‐induced impaired cell viability, enhanced cell apoptosis, and activated neuroinflammation were abrogated by the overexpression of miR‐140 in both PC12 and SH‐SY5S cells. Regarding the mechanisms underlying the function of miR‐410, phosphatase and tensin homolog (PTEN) was proposed as a direct target of miR‐410.

**Conclusion:**

All data revealed that serum downregulated miR‐410 in HIE serves as candidate diagnostic biomarker, and that miR‐410 exerts a neuroprotective role in OGD‐injured cells by improving cell viability and inhibiting cell apoptosis through targeting PTEN.

## Graphical abstract

MicroRNA‐410 (miR‐410) has been found to be deregulated in neonatal hypoxic‐ischemic encephalopathy (HIE). However, the clinical significance and biological function of miR‐410 remain largely elusive. This study aimed to investigate the expression and diagnostic performance of miR‐410 in HIE newborns, and explore the neuroprotective effect of miR‐410 in an oxygen‐glucose deprivation (OGD)‐induced cell injury model. All data revealed that serum downregulated miR‐410 in HIE serves as candidate diagnostic biomarker, and that miR‐410 exerts a neuroprotective role in OGD‐injured cells by improving cell viability and inhibiting cell apoptosis through targeting PTEN.

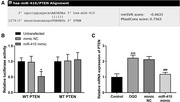

## 1 INTRODUCTION

Neonatal hypoxic‐ischemic encephalopathy (HIE) is a disorder with hypoxic and ischemic brain damages and mainly caused by perinatal asphyxia (Laptook, 2016). It is associated with high neonatal mortality and leads to long‐term neurologic morbidity (Glass, 2018). In developed countries, the incidence of HIE accounts for 1–8 per 1000 live newborns, and this data grow to 26 per 1000 in underdeveloped counties (Liu et al., 2017). To date, the therapeutic hypothermia is the only recognized treatment for neonatal HIE, but this strategy needs to be administrated within 6 h after birth, and its application in severe HIE cases is not ideal (Chiang et al., 2017). Thus, early diagnosis and efficient therapeutic approaches are urgently needed for newborns suffering from HIE.

MicroRNAs (miRNAs) are a series of small noncoding RNAs with important regulatory effects on a wide variety of biological processes, such as cell proliferation, migration, invasion, differentiation and apoptosis (Chao et al., 2018). Emerging evidence shows that human diseases are generally accompanied with dysregulation of miRNAs (Vishnoi and Rani, 2017). These aberrant miRNAs can serve as biomarkers for disease diagnosis and progression and participates disease pathogenesis (Bertoli et al., 2015). Some functional miRNAs have also been identified in neonatal HIE with pivotal roles in HIE development and progression, including miR‐210 (Ma et al., 2016), miR‐17‐5p (Chen et al., 2018), and miR‐204 (Chen et al., 2019). A recent study by O'Sullivan has investigated the altered miRNAs expression in umbilical cord blood in neonatal HIE, and microRNA‐410 (miR‐410) is one of the deregulated miRNAs in whole blood samples (O'Sullivan et al., 2019). In addition, miR‐410 has been documented to serve a protective role against brain injury in Parkinson's disease (Ge et al., 2019), anesthesia‐induced cognitive dysfunction (Su et al., 2019) and ischemic stroke (Liu et al., 2018). However, the clinical significance and biological function of miR‐410 in neonatal HIE remain largely elusive.

To improve the diagnosis of neonatal HIE, this study sought to evaluate the expression and diagnostic performance of miR‐410 in HIE newborns. In addition, this study constructed an oxygen and glucose deprivation (OGD)‐induced cell injury model in PC12 and SH‐SY5Y cells as the most commonly applied *in vitro* model for HIE (Zhang et al., 2019) to examine the biological function of miR‐410 in regulating neuronal cell survival.

## 2 MATERIALS AND METHODS

### 2.1 Study subjects and serum sample collection

One hundred and two HIE newborns were included in this study, who were collected from Weifang People's Hospital between May 2015 and April 2018. All the HIE newborns were full term infants without any brain injury caused by hyperbilirubinemia, intrauterine infection or severe brain dysplasia, and did not receive any therapy before sampling. The diagnosis of HIE was performed in accordance with the published criteria by Sarnat et al. (Sarnat and Sarnat, 1976) and confirmed with brain MRI results. In addition, 60 healthy newborns were collected as controls during a same time period, and none of them had medical history of perinatal asphyxia or disorder in nervous system after birth. According to the criteria by the Group of Neonatology et al. (Group of et al., 2005), the enrolled HIE newborns were grouped into mild, moderate and severe groups to facilitate further analyses. Femoral vein blood was collected from the newborns within 6 h after birth, then was centrifuged for the extraction of serum samples, which were stored at −80°C for further analyses. The protocols of this study were approved by the Ethics Committee of Weifang People's Hospital, and a written informed consent was obtained from each newborn's parent.

### 2.2 Cell culture and transfection

A rat pheochromocytoma cell line PC12 and a human neuroblastoma cell line SH‐SY5Y were purchased from the Cell Bank of Type Culture Collection of Chinese Academy of Sciences (Shanghai, China). The cells were cultured in Dulbecco's modified Eagle medium (DMEM; Invitrogen, Thermo Fisher Scientific, CA, USA) supplemented with 10% fetal bovine serum (FBS; Invitrogen, Thermo Fisher Scientific, CA, USA) at 37°C in a humidified incubator with 5% CO_2_. To regulate the expression of miR‐410 in neuronal cells, PC12 and SH‐SY5Y cells were transfected with miR‐410 mimic or mimic negative control (NC) by Lipofectamine 3000 (Invitrogen, Carlsbad, CA, USA) following the manufacturers’ protocols. The miR‐410 mimic and mimic NC sequences were synthesized by GenePharma (Shanghai, China).

### 2.3 Construction of oxygen‐glucose deprivation‐induced cell injury model

OGD experiment has been widely used to induce neuronal cell injury as an *in vitro* HIE model. In this study, PC12 and SH‐SY5Y cells were used and firstly cultured in glucose‐free medium at 37°C in a hypoxia incubator with 5% CO_2_, 94% N_2_, and 1% O_2_ for 2 h. Thereafter, the culture medium was replaced with growth medium containing glucose and incubated at 37°C under a normal condition with 5% CO_2_. The cells cultured under normal condition (37°C in a humidified incubator with 5% CO_2_) with DMEM supplemented 10% FBS were used as controls. For the cells with artificially regulated miR‐410, the OGD induction was conducted at 24 h after cell transfection.

### 2.4 RNA extraction and quantitative real‐time PCR

Total RNA in serum and cells was extracted using TRIzol reagent (Invitrogen, Carlsbad, CA, USA) and cDNA were synthesized from RNA by a PrimeScript RT reagent kit (TaKaRa, Shiga, Japan) as per the manufacturers’ instruction. Quantitative real‐time PCR (qRT‐PCR) was carried out to evaluate mRNA expression with the SYBR green I Master Mix kit (Invitrogen, Carlsbad, CA, USA) on a 7500 real‐time PCR system (Applied Biosystems, USA). The final relative expression value was calculated using the 2^−ΔΔCt^ method and normalized to cel‐miR‐39‐3p or GAPDH.

### 2.5 Cell viability analysis

Cell viability of PC12 and SH‐SY5Y was evaluated using MTT assay. Briefly, cells with a density of 5 × 10^3^ cell per well were seeded into 96‐well plates and cultured at 37°C with 5% CO_2_ for 24 h, then 0.5 mg mL^−1^ MTT solution was added into the cells for 4 h. After remove the MTT solution (Sigma‐Aldrich, MO, USA), 100 μL DMSO was added into the cells to dissolve the violet formazan crystals. The absorbance at 570 nm was measured to reflect the viability of PC12 and SH‐SY5Y cells.

### 2.6 Cell apoptosis analysis

Cell apoptosis was examined using flow cytometry analysis. Cells were collected and washed in cold PBS, then were resuspended in 200 μL binding buffer and stained with Annexin V‐FITC and propidium iodide (PI) at room temperature in the dark for 20 min, following the manufacturer's instruction of the FITC Annexin V/Dead Cell Apoptosis kit (Invitrogen, Carlsbad, CA, USA). The cell apoptosis rate was determined using a flow cytometer equipped with CellQuest Pro software (BD Bioscience).

### 2.7 Enzyme‐linked immunosorbent assay

To evaluate the inflammatory responses in the cell model, pro‐inflammatory cytokine levels were analyzed. Enzyme‐linked immunosorbent assay kits (R&D System, Abingdon, UK) were used to measure the levels of interleukin (IL)‐6 and tumor necrosis factor (TNF)‐α following the manufacturer's protocols.

### 2.8 Dual‐luciferase reporter assay

The miRanda bioinformatics software (www.microrna.org/microrna/home.do) was used to seek the putative target genes for miR‐410, and phosphatase and tensin homolog (PTEN) was found to possess the binding site of miR‐410 at its 3'‐UTR. A dual‐luciferase reporter assay was carried out to check the relationship between miR‐410 and PTEN. The wild‐type (WT) and mutant‐type (MT) 3'‐UTR of PTEN were separately cloned into the pmirGLO luciferase reporter vectors (Promega), and the combined vectors were co‐transfected into SH‐SY5Y cells with miR‐410 mimic or mimic NC using Lipofectamine 3000 (Invitrogen, Carlsbad, CA, USA). After 48 h of transfection, the relative luciferase of each group was measured using the Dual‐luciferase reporter assay system (Promega).

### 2.9 Statistical analysis

All the statistical analyses were performed with SPSS 21.0 software (SPSS Inc., Chicago, IL) and GraphPad Prism 7.0 software (GraphPad Software, Inc., USA). Quantitative data were presented as mean ± SD. Differences between groups were analyzed using Student's *t*‐test and one‐way ANOVA followed by Tukey's test. The receiver operating characteristic (ROC) curves were plotted to evaluate the clinical significance of miR‐410 in HIE newborns, and the area under the curve (AUC) was calculated to reflect the diagnostic accuracy of miR‐410. The results with *P* < .05 were considered statistically significant.

## 3 RESULTS

### 3.1 General characteristics of study newborns

The general characteristics of the enrolled newborns were recorded and listed in Table 1, which showed that there were no differences between the healthy and HIE newborns at gestation, birth weight, gender, and delivery method (all *P* > .05). Regarding the severity of HIE, the HIE newborns included 42 mild cases, 36 moderate cases, and 24 severe cases.

**TABLE 1:** Baseline characteristics of the collected newborns

**Characteristics**	**Health (*n* = 60)**	**HIE (*n* = 102)**	***P* value**
Gestation (mean ± SD, weeks)	39.9 ± 1.08	39.5 ± 1.34	0.120
Birth weight (mean ± SD, kg)	3.59 ± 1.00	3.35 ± 0.93	0.124
Gender (Male/Female)	30/30	54/48	0.718
Delivery method (Eutocia/Cesarean)	38/22	57/45	0.352
Severity (Mild/Moderate/Severe)	NA	42/36/24	NA

HIE, hypoxic‐ischemic encephalopathy.

### 3.2 Expression of microRNA‐410 in hypoxic‐ischemic encephalopathy newborns

**FIGURE 1:**
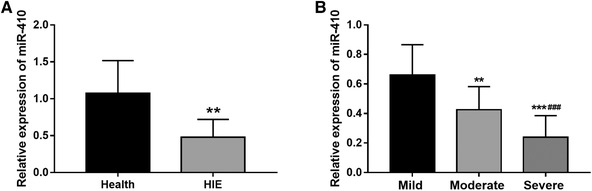
Expression of miR‐410 in HIE newborns. (A) Serum miR‐410 levels were reduced in HIE newborns compared with healthy controls (***P < .001). (B) Serum miR‐410 levels were decreased along with the increase of severity of HIE (**P < .01, ***P < .001 vs. Mild; ^###^ *P* < .001 vs. Moderate)

Serum levels of miR‐410 were examined using qRT‐PCR. The analysis results shown in Figure 1(A) indicated that HIE newborns had lower serum miR‐410 expression than the healthy controls (*P* < .01). By comparing miR‐410 expression in HIE newborns with different severity, the highest expression was observed in mild HIE group, followed by the moderate group, and the lowest expression was presented in serve HIE group (*P* < .01, Figure 1(B)).

### 3.3 Clinical significance of microRNA‐410 in neonatal hypoxic‐ischemic encephalopathy diagnosis

**FIGURE 2:**
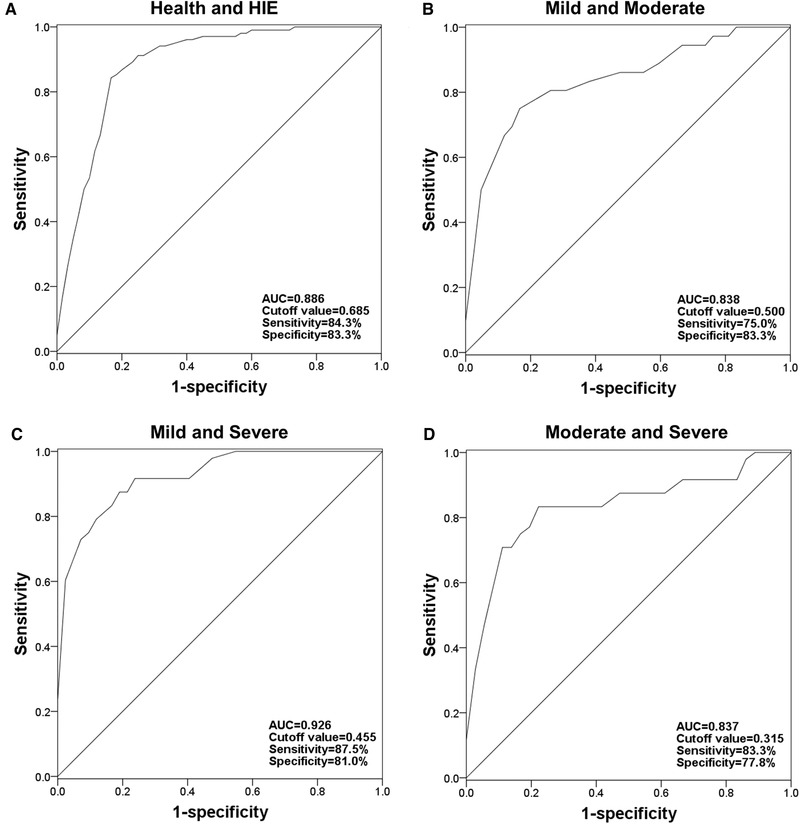
Diagnostic significance of serum miR‐410 in HIE newborns. (A) A ROC for serum miR‐410 between healthy and HIE newborns. (B) A ROC for serum miR‐410 between mild and moderate HIE. (C) A ROC for serum miR‐410 between mild and severe HIE. (D) A ROC for serum miR‐410 between moderate and severe HIE. AUC, area under the curve

Given the deregulated expression of miR‐410 in HIE newborns, its diagnostic performance was evaluated using a ROC curve. As shown in Figure 2(A), a ROC curve with an AUC of 0.886 was plotted based on serum miR‐410 for HIE newborns, and the sensitivity and specificity were 84.3% and 83.3%, respectively, at a cutoff value of 0.685. Furthermore, the clinical significance of miR‐410 to predict the progression of HIE was assessed by the ROC curves for HIE cases with different severity. The results shown in Figure 2(B)–(D) indicated that serum miR‐410 had diagnostic accuracy for the differentiation between mild and moderate HIE (AUC = 0.838), mild and severe HIE (AUC = 0.926), and moderate and severe HIE (AUC = 0.837).

### 3.4 Downregulated expression of microRNA‐410 in oxygen‐glucose deprivation‐induced cell injury model

**FIGURE 3:**
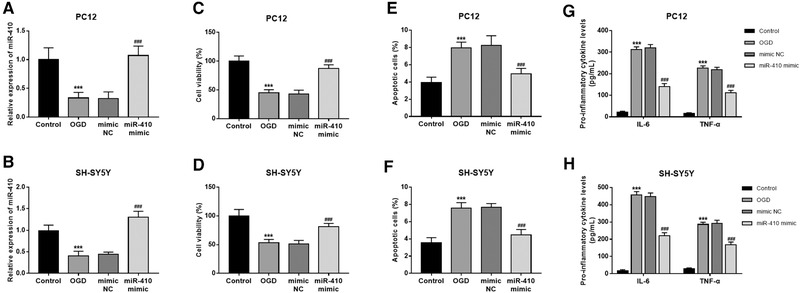
Effect of miR‐410 on cell viability and apoptosis in OGD‐injured PC12 and SH‐SY5Y cells. (A),(B) Expression of miR‐410 in OGD‐injured cell models. (C),(D) The inhibited cell viability induced by OGD was abrogated by the overexpression of miR‐410. (E),(F) The OGD‐induced increase in cell apoptosis rate was inhibited by the upregulation of miR‐410. (G),(H) The increased IL‐6 and TNF‐α levels induced by OGD experiment were decreased by the overexpression of miR‐410. ***P < .001 vs. Control; ^###^ *P* < .001 vs. OGD

An *in vitro* HIE model was constructed by OGD treatment in PC12 and SH‐SY5Y cells. The expression of miR‐410 was significantly reduced in both the OGD‐injured PC12 and SH‐SY5Y cells (both *P* < .001, Figure 3(A),(B)).

### 3.5 MicroRNA‐410 significantly ameliorates oxygen‐glucose deprivation‐induced cell injury and inflammatory responses

The neuronal cell injury was measured by analyzing cell viability and apoptosis. As shown in Figure 3(C)–(F), the OGD treatment in PC12 and SH‐SY5Y cells led to decreased cell viability and increased cell apoptosis rate (all *P* < .05). A gain‐of‐function analysis was performed by cell transfection with miR‐410 mimic, which resulted in the significantly increased miR‐410 expression in the cell injury model (*P* < .001, Figure 3(A),(B)). By overexpression of miR‐410, the OGD‐induced neuronal cell injury was remarkably attenuated, that indicated by the elevated cell viability and inhibited cell apoptosis in OGD‐injured cell model (all *P* < .05, Figure 3(C)–(F)). In addition, the activated inflammatory responses induced by OGD treatment were significantly eliminated by the upregulation of miR‐410 in both PC12 and SH‐SY5Y cells, which manifested by the reduced IL‐6 and TNF‐α levels (all *P* < .05, Figure 3(G),(H)).

### 3.6 Phosphatase and tensin homolog is directly regulated by microRNA‐410

**FIGURE 4:**
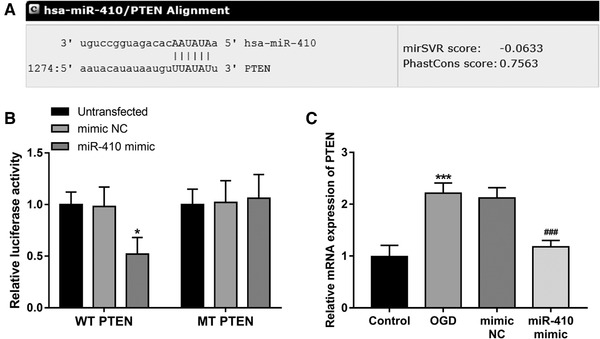
miR‐410 directly targeted PTEN in the OGD‐induced SH‐SY5Y cell injury model. (A) The binding site of miR‐410 and PTEN. (B) The directly interaction between miR‐410 ad PTEN evidenced by the luciferase activity results (*P < .05 vs. Untransfected). (C) Overexpression of miR‐410 led to significantly decreased PTEN mRNA expression in the OGD‐induced SH‐SY5Y cell injury model (***P < .001 vs. Control; ^###^ *P* < .001 vs. OGD)

By bioinformatics analysis, PTEN was predicted as a potential target gene of miR‐410, and the alignment of the seed regions of miR‐410 with the 3'‐UTE of PTEN was shown in Figure 4(A). The results of subsequent luciferase reporter assay revealed that the overexpression of miR‐410 could markedly reduce the luciferase activity of a reporter vector containing the WT 3'‐UTE of PTEN (*P* < .05, Figure 4(B)), but did not affect the luciferase activity results for the vector containing MT 3'‐UTE of PTEN (*P* > .05). Furthermore, the mRNA expression of PTEN in the OGD‐induced SH‐SY5Y cell injury model was analyzed, which showed that the OGD treatment could significantly increase the mRNA expression of PTEN (*P* < .01, Figure 4(C)), while this effect was remarkable abrogated by the overexpression of miR‐410 (*P* < .01).

## 4 DISCUSSION

Emerging studies have demonstrated that miRNAs serve important roles in the development and progression of brain injury‐related diseases (Sarkar et al., 2019). For example, Xi et al. have reported that intracerebral hemorrhage patients had reduced expression of miR‐27a‐3p, and the overexpression of miR‐27a‐3p could significantly attenuate blood‐brain barrier disruption and brain injury (Xi et al., 2018). The increased levels of miR‐124‐3p in microglial exosomes have been documented to be involved in the progression of traumatic brain injury through regulating inflammation and neuronal cell viability (Huang et al., 2018). Shi et al. found that miR‐155‐5p was elevated in middle cerebral artery occlusion/reperfusion rats and served as a potential therapeutic target for ischemic stroke by modulating neuronal cell apoptosis (Shi et al., 2020). These aforementioned studies indicate that brain injury‐associated diseases are generally accompanied with aberrant miRNAs, which may act pivotal roles in disease pathogenesis.

In neonatal HIE, there are also some functional miRNAs. A study by Zhang et al. documented that miR‐200b‐3p was elevated in neonatal HIE rats and the inhibition of miR‐200b‐3p had a beneficial effect on the brain damage (Zhang et al., 2020). The upregulated expression of miR‐210 has also been reported in neonatal HIE and was considered as a therapeutic target by regulating microglia‐mediated neuroinflammation (Li et al., 2020). The decreased miR‐410 has been reported in a study by O'Sullivan et al., which focused on the miRNAs with abnormal expression patterns in neonatal HIE (O'Sullivan et al., 2019). In this study, serum downregulated levels of miR‐410 were also observed in HIE newborns compared with healthy controls. In addition, the expression of miR‐410 was significantly distinct in HIE newborns with different severity. Previous studies have provided evidence for miR‐410 to participate the development of brain injury in some diseases (Ge et al., 2019, Su et al., 2019, Liu et al., 2018). Thus, we suspected that miR‐410 might serve a potential role in the development and progression of neonatal HIE.

Considering the aberrant expression of miR‐410 in serum samples of HIE newborns, its clinical significance was further evaluated. miRNAs are considered a group of good diagnostic tools for various human diseases, owing to their stability and detectability in serum samples (Hayes et al., 2014). Some miRNAs with deregulated expression levels have been proposed as biomarkers for neonatal HIE diagnosis or prognosis, such as miR‐210 and miR‐374a (Wang et al., 2018). In this study, the diagnostic value of serum miR‐410 was evaluate using ROC analysis, and the results suggested that serum miR‐410 had relatively high diagnostic accuracy for screening HIE newborns from healthy controls. In addition, given the significant differences in miR‐410 levels between HIE neonates with different severity, the discriminating potential of miR‐410 for neonatal HIE severity was investigated. The ROC curves indicated that miR‐410 could be used as biomarker to predict the severity of neonatal HIE. However, the sample size in our study was finite, which might limit the result accuracy. Thus, further investigations are necessary to confirm our conclusion with a large‐scale study population.

To further understand the functional role of miR‐410 in neonatal HIE pathogenesis, this study established an *in vitro* HIE model using the OGD‐injured PC12 and SH‐SY5Y cells. The expression of miR‐410 was as expected to be significantly downregulated after OGD treatment in neonatal cells. In addition, the neuronal cell injury induced by OGD, that manifested by the inhibited cell viability and enhanced cell apoptosis, was remarkably attenuated by the overexpression of miR‐410, which indicated the neuroprotective role of miR‐410 in OGD‐induced cell injury model. Neuroinflammation also plays critical role in the progression of HIE, aggravating neuronal cell injury (Li et al., 2020). In the OGD‐induced cell injury model, the levels of pro‐inflammatory cytokines were significantly elevated, indicating the activated inflammatory responses. By overexpressing the expression of miR‐410 in the cell model, the upregulated pro‐inflammatory cytokines were remarkably inhibited, which suggested that miR‐410 overexpression could relieve the inflammation induced by OGD in both PC12 and SH‐SY5Y cells. Therefore, it is concluded that miR‐410 might exert neuroprotective effects by ameliorating neuronal viability and restraining inflammatory responses. The neuroprotection of miR‐410 has been previously documented in Parkinson's disease and ischemic stroke (Ge et al., 2019, Liu et al., 2018). These published evidence combined our analysis results led us to deduce that the methods to upregulate miR‐410 might be novel therapeutic strategies for the treatment of HIE.

This study predicted the putative target genes of miR‐410, and analyzed the relationship of PTEN, which was one of the predicted targets with important role in neuroprotection, with miR‐410 in the OGD‐induced cell injury model. PTEN is an important upstream regulator of the AKT signaling pathway and its inhibition can prevent neuron injury after hypoxia‐ischemia (Zhao et al., 2013). In neonatal HIE, PTEN has been documented to mediate the protective effect of sevoflurane post‐conditioning against brain injury (Xue et al., 2019). In addition, the neuroprotective role of miR‐410 has been reported in Parkinson's disease by targeting PTEN (Ge et al., 2019). In this study, an increase in the mRNA expression of PTEN was obtained in the OGD‐induced cell injury model, and this effect was significantly attenuated by miR‐410, which inspired us that miR‐410 in neonatal HIE might also exert neuroprotection by targeting PTEN. However, the molecular mechanisms underlying the function of miR‐410 need to be further explored regarding the related signaling pathways. In addition, another limitation of this study is the lack of *in vivo* animal experiments to confirm the mechanisms of miR‐410. Thus, further explorations using neonatal HIE animal models are necessary to further uncover the role and mechanisms of miR‐410 in neonatal HIE.

Taken together, our analysis results reveal that serum reduced miR‐410 may serve as a biomarker for the diagnosis and progression of neonatal HIE, and the overexpression of miR‐410 exerts a neuroprotective role in an OGD‐induced cell injury model. The findings of this study provide a novel insight in the clinical and functional role of miR‐410 in neonatal HIE, and the methods to increase miR‐410 may provide new ideas to improve the therapeutic approaches for HIE treatment.

## CONFLICTS OF INTEREST

The authors have declared no conflict of interest.

## FUNDING

A Research Project of Weifang (WFWSJK‐2020‐197).

## Data Availability

The data used to support the findings of this study are available from the corresponding author upon reasonable request.
